# Ongoing Transmission of Hepatitis B Virus in Rural Parts of the Netherlands, 2009–2013

**DOI:** 10.1371/journal.pone.0117703

**Published:** 2015-02-23

**Authors:** Loes C. Soetens, Birgit H. B. van Benthem, Anouk Urbanus, Jeroen Cremer, Kimberly S. M. Benschop, Ariene Rietveld, Erik I. van Dijk, Susan J. M. Hahné

**Affiliations:** 1 Epidemiology and Surveillance Unit, Centre of Infectious Disease Control, National Institute for Public Health and the Environment, Bilthoven, The Netherlands; 2 National Coordination Centre for Communicable Disease Control, Centre of Infectious Disease Control, National Institute for Public Health and the Environment, Bilthoven, The Netherlands; 3 Infectious Diseases Research, Diagnostics and Screening, Centre of Infectious Disease Control, National Institute for Public Health and the Environment, Bilthoven, The Netherlands; 4 Department of Infectious Disease Control, Municipal Health Service Hart voor Brabant, ‘s-Hertogenbosch, The Netherlands; 5 Department of Infectious Disease Control, Municipal Health Service Drenthe, Assen, The Netherlands; Kliniken der Stadt Köln gGmbH, GERMANY

## Abstract

**Background:**

Reported acute hepatitis B incidence in the Netherlands reached its nadir in 2013. However, regional signals about increased number of hepatitis B cases raised the question how hepatitis B incidence was distributed over the country. In this study, regional differences in hepatitis B epidemiology were investigated using epidemiological and molecular data.

**Methods:**

Acute hepatitis B virus (HBV) infections, reported between 2009–2013, were included. If serum was available, a fragment of S and C gene of the HBV was amplified and sequenced. Regional differences in incidence were studied by geographical mapping of cases and cluster analysis. Regional differences in transmission were studied by constructing regional maximum parsimony trees based on the C gene to assess genetic clustering of cases.

**Results:**

Between 2009 and 2013, 881 cases were notified, of which respectively 431 and 400 cases had serum available for S and C gene sequencing. Geographical mapping of notified cases revealed that incidences in rural border areas of the Netherlands were highest. Cluster analysis identified two significant clusters (p<0.000) in the South-western and North-eastern regions. Genetic cluster analysis showed that rural border areas had relatively large clusters of cases with indistinguishable sequences, while other regions showed more single introductions.

**Conclusion:**

This study showed that regional differences in HBV epidemiology were present in the Netherlands. Rural border regions showed higher incidences and more ongoing transmission, mainly among MSM, than the more urban inland areas. Therefore, preventive measures should be enhanced in these regions.

## Introduction

Hepatitis B virus (HBV) infection can cause a broad spectrum of disease outcomes from asymptomatic infection to severe complications. People with chronic HBV infection are at increased risk of developing hepatic decompensation, cirrhosis and hepatocellular carcinoma. Since the introduction of hepatitis B vaccination in the 1980s, the incidence of hepatitis B infection dropped tremendously worldwide [1,2]. However, in specific high-risk groups, the HBV prevalence and incidence can still be substantial [2].

In the Netherlands, reported acute hepatitis B incidence decreased since 2004 and reached its nadir in 2013 with 0.8 per 100,000 population [3]. This incidence was comparable with the overall acute hepatitis B incidence reported in the European Union/European Economic Area and with the incidence reported in the neighbouring country Germany in 2011 [4]. The most common route of transmission to acquire hepatitis B virus infection in the Netherlands is through sexual contact, mainly by men who have sex with men (MSM)[5], whereas in Europe in general the most common route of transmission is through heterosexual sexual contact [4].

In 2002, the Netherlands adopted a selective vaccination programme targeted at behavioural high-risk groups. The programme currently offers free vaccination to MSM and commercial sex workers (CSW). Vaccination takes place at Public Health Service (PHS) centres, sexually transmitted infections (STI) clinics and outreach locations. This selective vaccination programme has been proven effective, especially in reducing hepatitis B infections among MSM [5–7]. Since 2011, hepatitis B vaccination is part of the national immunisation programme, which means that all infants are offered vaccination against hepatitis B.

Although the national incidence of reported acute HBV infection in the Netherlands is decreasing, in the last years some regional PHS centres observed significant increases in the incidence, suggesting hepatitis B incidence was not distributed evenly throughout the country. This could have implications for the targeting of preventive and control measures. We therefore investigated regional differences in hepatitis B epidemiology by studying notifications, molecular data of their hepatitis B viruses and data of the vaccination programmes for behavioural high risk groups, in order to inform hepatitis B prevention.

## Methods

### Data collection

In the Netherlands, physicians and laboratories are obliged to notify acute hepatitis B infections. The case definition for reporting is a positive laboratory result for immunoglobulin M antibody to the hepatitis B core antigen (IgM anti-HBc) and/or hepatitis B surface antigen (HBsAg; the latter only after exclusion of hepatitis A and C viruses) in a person with an acute onset of symptoms compatible with acute hepatitis and with jaundice and/or increased serum aminotransferase. To study regional differences in the incidence of acute hepatitis B we used data from the National Register for Notifiable Diseases (‘Osiris’). We used all cases diagnosed and registered between 2009 and 2013. The (molecular) epidemiology of cases from 2004–2010, was shown reported previously [5]. Of these cases, epidemiological information was collected, among which age, sex, postal code, date of diagnosis and (probable) mode of transmission. Data were extracted from the web-based notification register in March 2014. All information was available on an individual level.

To study regional differences in transmission, we used molecular typing data collected by the National Institute for Public Health and the Environment (RIVM). Since 2004, medical microbiology laboratories are asked to submit serum samples of patients diagnosed with acute hepatitis B to the laboratory of the RIVM. We used all available sequences of acute hepatitis B infections reported in 2009–2013 to conduct a genetic cluster analysis as described below. All sequences used in our analysis are deposited in GenBank (accession numbers: C-gene, KP243201—KP243600; S-gene, KP243601—KP244031).

To examine regional differences in preventive activities regarding hepatitis B infection, we included data of the national vaccination programme for behavioural high-risk groups in this study. The number of vaccinations and percentage of vaccinations performed by outreach activities, recorded between 2009–2013, were used. We also calculated the rate of first vaccinations per 100,000 men or women (depending on the risk group) for each region over 5 years. Data on population size by region from Statistic Netherlands [8] were used to calculate this rate. Data of the vaccination programme were available on regional level.

Statistical differences in categorical variables across regions, spatio-temporal clusters or transmission routes were calculated using the Chi-squared test, and statistical differences in medians across regions or transmission routes were calculated using the Kruskal-Wallis test. All statistical analyses were performed using the SAS 9.3 statistical program.

Ethical approval was sought but deemed not necessary by the ethics committee of the Amsterdam Medical Centre. The reason for this was that residual sera collected for HBV diagnostic purposes were in this project used anonymously for HBV testing only. Informed consent by participants was therefore not part of the study procedures.

### Geographic cluster analysis

**Fig 1:**
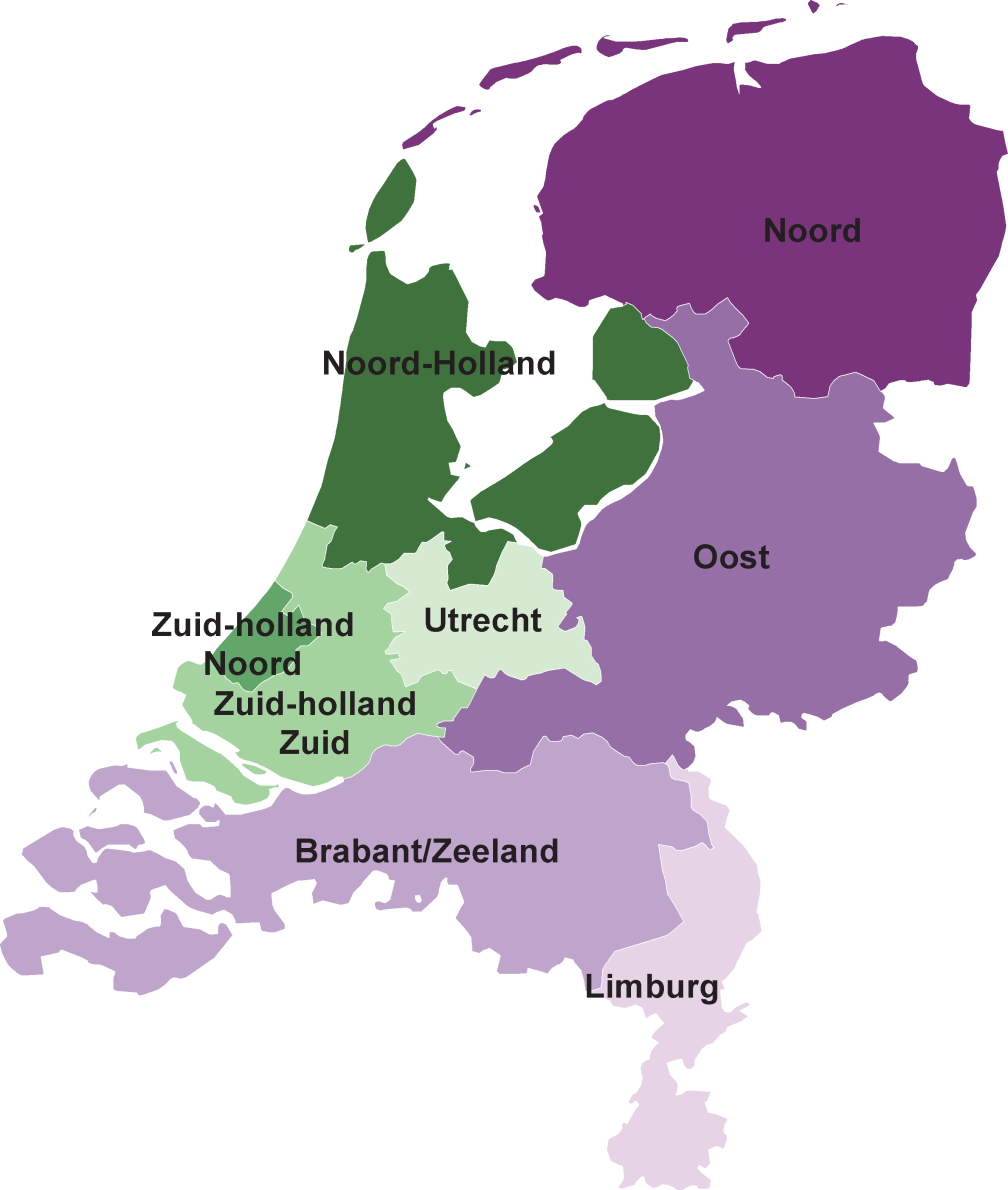
Regions for sexual health care in the Netherlands. Purple areas indicate the more rural regions, green areas indicate the more urban regions.

To study regional differences in epidemiology of acute hepatitis B, we stratified statistical analyses by region (Fig. 1). Furthermore, all cases were mapped using the geographic information system ArcGIS software, version 10.1. The postal code was used to identify the geographic location of cases, but data were aggregated and analysed on municipality level for privacy reasons. Incidences per municipality were calculated per 100,000 population. To identify geographic clusters of cases in the period 2009–2013, cluster analysis on municipality level was performed using the SaTScan software version 9.0 [9]. We used a discrete Poisson model, which assumes that the number of cases in each location is Poisson-distributed, and analysed the data with the space-time scan statistic to identify clusters in space and time. The space-time scan statistic uses cylindrical search windows in a space-time cube in order to seek significant spatio-temporal clusters. We set SaTScan to scan only for areas with high rates, that is for clusters.

### Genotyping and phylogenetic analysis

The RIVM amplified and sequenced fragments of the S- and C-gene (656-nucleotide (nt) and 655-nt respectively) as described in previous studies [5,10,11]. The S-gene was used for typing [12], while the C-gene was used to study transmission chains; an earlier study [11] showed that the S-gene, a more conserved region, is more useful for distinguishing genotypes than the C-gene. The latter, however, is preferable for identifying recent transmission chains, due to the relatively high mutation rate in this region. C-gene sequences were aligned using the software Bionumerics version 7.1 and were used to construct phylogenetic trees with the maximum parsimony method, to assess genetic clustering of cases. A cluster was defined as three or more cases with indistinguishable sequences. Patients with indistinguishable sequences were displayed in the same node in the maximum parsimony tree (the size of the node is relative to the number of indistinguishable sequences). The trees were categorized according to different geographic regions shown in Fig. 1 and probable mode of transmission.

## Results

### Study population

Between 2009 and 2013, 881 cases were notified with acute hepatitis B in the Netherlands. The basic characteristics of the study population by region are shown in Table 1. The five-year incidence varied between 0.73 and 1.57 infections per 100,000 population across the regions, and was in all regions higher in men than in women. Across the regions, the percentage of men varied between 65.5% and 83.7%, the percentage of patients born in the Netherlands ranged from 65.1% to 92.3% and the median age varied between 32 years and 47 years. Considering the most probable mode of transmission, the country can be divided in regions where most patients got infected through heterosexual contact (Noord-Holland, Utrecht and Zuid-Holland Noord; in general the more urban areas) and regions where most patients got infected through male homosexual contact or were men with an unknown mode of transmission (Brabant/Zeeland, Zuid-Holland Zuid, Limburg, Noord and Oost; mainly the more rural areas of the Netherlands).

**Table 1:** Basic characteristics of acute hepatitis B patients by region of reporting, notified between 2009–2013 in the Netherlands.

	Brabant/Zeeland	Limburg	Noord	Noord-Holland	Oost	Utrecht	Zuid-Holland Noord	Zuid-Holland Zuid		Total
	n = 173	n = 52	n = 135	n = 145	n = 129	n = 45	n = 63	n = 139		n = 881
	N(%)	N(%)	N(%)	N(%)	N(%)	N(%)	N(%)	N(%)	*P*-value	N(%)
Incidence per 100,000*										
Total	1.22	0.93	1.57	0.94	0.82	0.73	1.23	1.11	0.000	1.06
Men	1.99	1.55	2.65	1.25	1.32	1.13	1.93	1.74	0.000	1.66
Women	0.45	0.32	0.51	0.64	0.33	0.35	0.54	0.50	0.128	0.47
Sex										
Men	141 (81.5)	43 (82.7)	113 (83.7)	95 (65.5)	103 (79.8)	34 (75.6)	49 (77.8)	107 (77.0)	0.012	685 (77.8)
Women	32 (18.5)	9 (17.3)	22 (16.3)	50 (34.5)	26 (20.2)	11 (24.4)	14 (22.2)	32 (23.0)		196 (22.3)
Age										
Median	42.5	45.0	45.0	38.0	47.0	32.0	38.0	45.0	<0.0001	43.0
(IQR)	(33.0–51.0)	(34.5–57.5)	(30.0–54.0)	(28.0–47.0)	(31.0–60.0)	(25.0–45.0)	(27.0–52.0)	(34.0–57.0)		(30.0–53.5)
Ethnicity										
Dutch	139 (80.4)	47 (90.4)	113 (83.7)	103 (71.0)	119 (92.3)	35 (77.8)	41 (65.1)	109 (78.4)	<0.0001	706 (80.1)
Non-Dutch	34 (19.6)	5 (9.6)	22 (16.3)	42 (29.0)	10 (7.7)	10 (22.2)	22 (34.9)	30 (21.6)		175 (19.9)
Transmission route										
Heterosexual men	32 (18.5)	8 (15.4)	19 (14.1)	35 (24.1)	22 (17.1)	13 (28.9)	19 (30.2)	33 (23.7)	0.004	181 (20.5)
MSM	49 (28.3)	18 (34.6)	42 (31.1)	35 (24.1)	32 (24.8)	9 (20.0)	12 (19.1)	42 (30.2)		239 (27.1)
Women—sex. contact	24 (13.9)	7 (13.5)	16 (11.9)	38 (26.2)	19 (14.7)	9 (20.0)	10 (15.9)	23 (16.6)		146 (16.6)
Other	16 (9.3)	4 (7.7)	5 (3.7)	7 (4.8)	13 (10.1)	3 (6.7)	9 (14.3)	14 (10.1)		71 (8.1)
Unknown-men	48 (27.8)	14 (26.9)	47 (34.8)	23 (15.9)	39 (30.2)	10 (22.2)	9 (14.3)	22 (15.8)		212 (24.1)
Unknown-women	4 (2.3)	1 (1.9)	6 (4.4)	7 (4.8)	4 (3.1)	1 (2.2)	4 (6.4)	5 (3.6)		32 (3.6)
Number sequenced										
S-gene	79 (45.7)	41 (78.8)	91 (67.4)	53 (36.6)	74 (57.4)	21 (46.7)	25 (39.7)	47 (33.8)	<0.0001	431 (48.9)
Genotype based on S-gene										
A	62 (78.5)	29 (70.7)	80 (87.9)	27 (50.9)	56 (75.7)	11 (52.4)	12 (48.0)	31 (66.0)	0.000	308 (71.5)
B	2 (2.5)	0 (0.0)	1 (1.1)	3 (5.7)	2 (2.7)	1 (4.8)	0 (0.0)	2 (4.3)		11 (2.6)
C	4 (5.1)	3 (7.3)	3 (3.3)	2 (3.8)	4 (5.4)	3 (14.3)	3 (12.0)	5 (10.6)		27 (6.3)
D	5 (6.3)	4 (9.8)	4 (4.4)	18 (34.0)	8 (10.8)	6 (28.6)	7 (28.0)	6 (12.8)		58 (13.5)
E	5 (6.3)	2 (4.9)	2 (2.2)	2 (3.8)	4 (5.4)	0 (0.0)	2 (8.0)	2 (4.3)		19 (4.4)
F	1 (1.3)	3 (7.3)	1 (1.1)	1 (1.9)	0 (0.0)	0 (0.0)	1 (4.0)	1 (2.1)		8 (1.9)

*The incidence was calculated as the average over the yearly incidences in 2009–2013

### Spatio-temporal clusters

**Fig 2:**
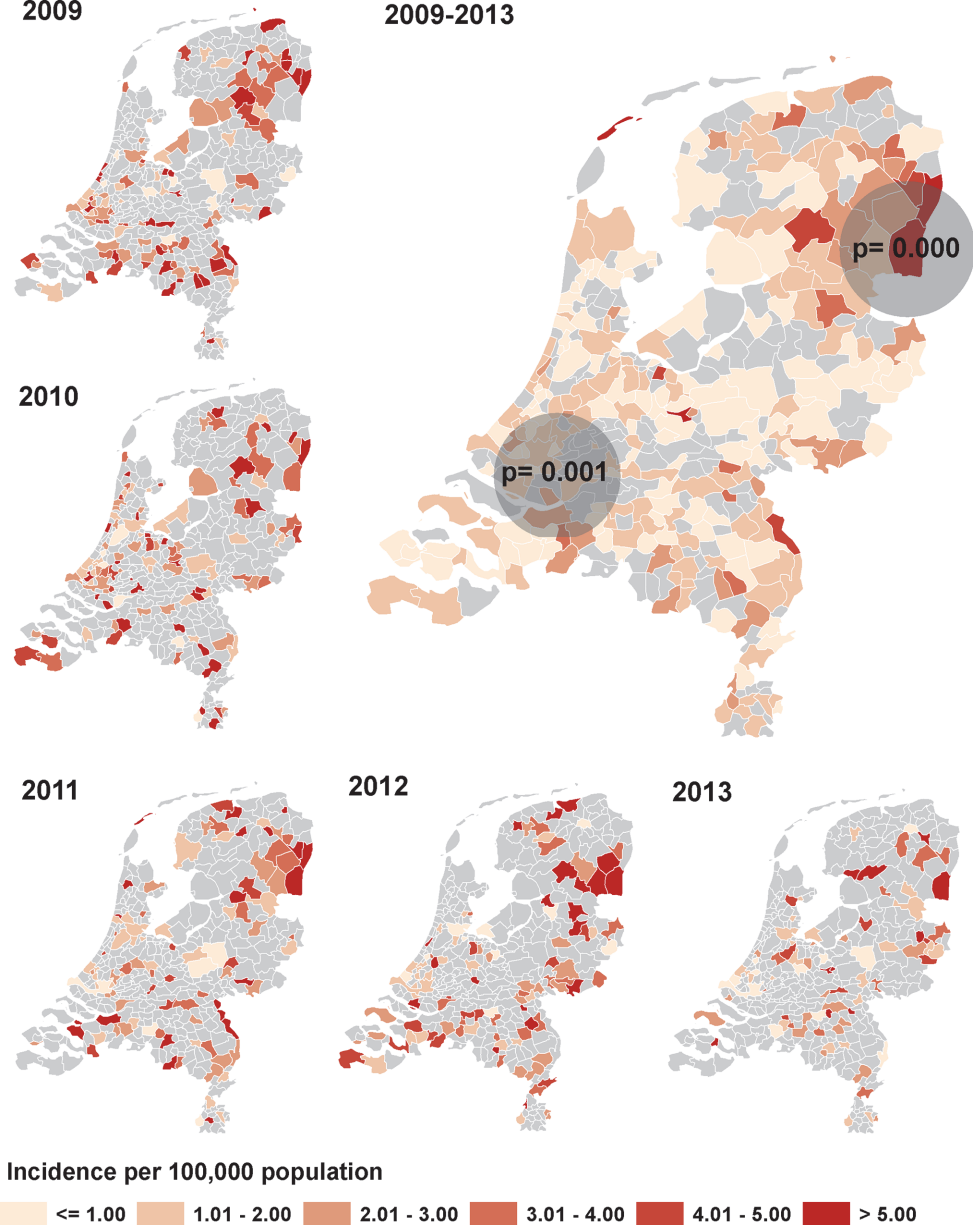
Incidence of acute HBV in the Netherlands, by year and overall incidence 2009–2013. The dark grey circles indicate regions with significant geographic clusters.

In Fig. 2, the geographical distribution of the HBV incidence over time is shown. The highest incidences in the separate years and overall were seen in the north-eastern and southern regions of the country, while most large cities are in the central and western regions of the country. The space-time scan statistic identified two significant clusters. The first cluster was present between February 2009 and December 2010, and was located in the south-western region of the country (n = 96, *p* = 0.001). The second cluster was present between November 2010 and April 2013, and was located in the north-eastern region of the country (n = 39, *p* = 0.000). When comparing the patients inside and outside the spatio-temporal clusters (Table 2), it can be observed that the patients in the south-western spatio-temporal cluster did not differ from the patients outside the clusters with respect to sex, median age, ethnicity and transmission route (*p*> 0.05). However, the patients within the north-eastern spatio-temporal cluster were generally more often male (*p* = 0.021) and were more likely to be MSM or men with an unknown mode of transmission (*p* = 0.003) than patients outside the spatio-temporal clusters.

**Table 2:** Characteristics of patients within the spatio-temporal clusters compared to patients outside the spatio-temporal clusters.

	Not in spatio-temporal cluster (ref)	Southwestern spatio-temporal cluster		Northeastern spatio-temporal cluster	
	n = 746	n = 96		n = 39	
	n (%)	n (%)	p-value*	n (%)	p-value*
Sex					
Men	570 (76.4)	79 (82.3)	0.197	36 (92.3)	0.021
Women	176 (23.6)	17 (17.7)		3 (7.7)	
Age					
Median (IQR)	42 (30–53)	43 (29.5–53.5)	0.937	46 (42–54)	0.057
Ethnicity					
Dutch	594 (79.6)	75 (78.1)	0.403	37 (94.9)	0.053
Non-Dutch	126 (16.9)	15 (15.6)		1 (2.6)	
Unknown	26 (0.1)	6 (6.3)		1 (2.6)	
Transmission route					
Heterosexual men	155 (20.8)	24 (25.0)	0.096	2 (5.1)	0.003
MSM	198 (26.5)	28 (29.2)		13 (33.3)	
Women—sex. contact	127 (17.0)	16 (16.7)		3 (7.7)	
Other	57 (7.6)	12 (12.5)		2 (5.1)	
Unknown—men	177 (23.7)	16 (16.7)		19 (48.7)	
Unknown—women	32 (4.3)	0 (0.0)		0 (0.0)	
Number sequenced					
C-gene	343 (46.0)	28 (29.2)	0.002	27 (69.2)	0.005
Median cluster size (IQR)					
C-gene	7 (1–54)	3.5 (1–51)	0.901	51 (51–70)	<0.0001
Genetic cluster ≥ 3					
C-gene	193 (56.3)	16 (57.1)	0.929	23 (85.2)	0.003

*Compared to patients not in a spatio-temporal cluster.

### Genetic clusters

**Fig 3:**
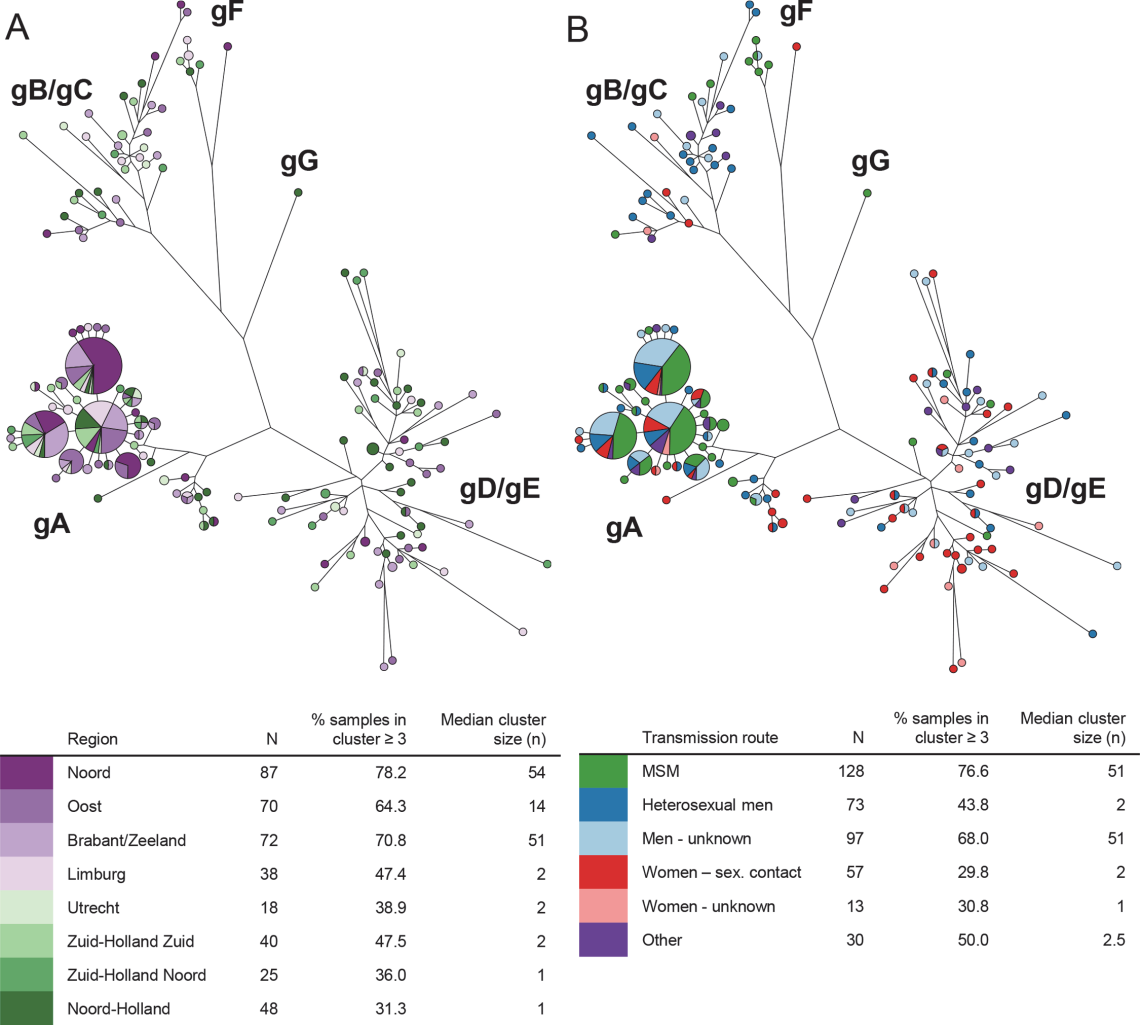
Maximum parsimony trees of HBV cases (2009–2013) in the Netherlands based on C-gene sequences (N = 400), by region (Fig. 3A) and most probable mode of transmission (Fig. 3B). (3A: purple = rural areas, green = urban areas; Genotypes: gA = genotype A, gB = genotype B, gC = genotype C, gD = genotype D, gE = genotype E, gF = genotype F, gG = genotype G; patients with indistinguishable sequences are displayed together in one node and the size of the node is relative to the number of indistinguishable sequences)

Of the 881 notified cases, 492 samples (56%) arrived at the RIVM for molecular typing. Reasons why samples did not arrive at the RIVM were refused cooperation by the patient (n = 101), insufficient or no material available in the local laboratory (n = 102), refused cooperation of the local laboratory (n = 50) and unknown (n = 136). In the end, 431 sequences (87.6%) of the S-gene and 400 sequences (81.3%) of the C-gene were successfully typed. The percentage samples sent in for sequencing differed by region (ranging from 33.8% in Zuid-Holland Zuid to 78.8% in Limburg, *p*< 0.0001, Table 1) and between the reported mode of transmission (ranging from 40.6% women with an unknown mode of transmission to 58.2% in MSM, *p* = 0.015). However, the transmission mode distribution of the received samples did not differ by region (*p* = 0.903), suggesting that the difference in received samples between the regions was not due to selection in a certain risk group, but was mainly due to external factors, such as mentioned above. Of all successfully typed S-gene sequences (n = 431), 71.5% was genotype A, 2.6% was genotype B, 6.3% was genotype C, 13.5% was genotype D, 4.4% was genotype E and 1.9% was genotype F (Table 1). Across all regions, genotype A was the most frequently reported genotype, followed by genotype D. The maximum parsimony trees based on C-gene of all HBV cases in the Netherlands, reported between 2009 and 2013, are shown in Fig. 3. In Fig. 3A, the cases are coloured by region of reporting and cases with indistinguishable sequences are clustered together in nodes. The percentage of patients present in a cluster of 3 or more individuals varied greatly between the regions, ranging from 31.3% to 78.2% (*p*< 0.0001). Similarly, the median cluster size varied as well between the regions, ranging from 1 to 54 (*p*< 0.0001).

In Fig. 3B the same maximum parsimony tree is shown as in Fig. 3A, but now the tree is coloured by most probable mode of transmission. In this figure, it can be seen that cases in a cluster were more likely to be MSM and men with an unknown mode of transmission (higher percentage of samples present in a cluster of 3 or more samples and higher median cluster sizes).

### Genetic clustering within the spatio-temporal clusters

To assess if patients within a spatio-temporal cluster also showed higher genetic clustering rates, we compared the percentage of patients in a genetic cluster of 3 or more samples and the median cluster size of patients within a spatio-temporal cluster to patients outside a spatio-temporal cluster (Table 2). Compared to the patients outside a spatio-temporal cluster, patients within the south-western spatio-temporal cluster had significantly less samples available for sequencing (*p* = 0.002), but the percentage of patients in a cluster or the median cluster size did not significantly differ from those outside a spatio-temporal cluster. Patients within the north-eastern spatio-temporal cluster had significantly more samples available for sequencing compared to the patients outside the spatio-temporal cluster (*p* = 0.005). In addition, the percentage of patients in a cluster of three or more was significantly higher (*p* = 0.003) and the median cluster size was larger (*p*< 0.0001) within this spatio-temporal cluster than outside the spatio-temporal cluster.

### Preventive activities

The number and location of first vaccinations (2009–2013) of the selective HBV vaccination programme for behavioural risk-groups are displayed in Table 3. Of all first vaccinations given within the HBV vaccination programme, most vaccinations were received by MSM, compared to CSW, varying from 54.3 to 80.7% of the total number of vaccinations across the regions. We calculated the number of first vaccinations received by MSM per 100,000 men for each region, which varied greatly across the regions: from 112 to 425 vaccinations per 100,000 men over 5 years. It is worth noticing that the region with the lowest vaccination rate among MSM reported the highest incidence among men (Table 1). We calculated the same rate for the first vaccinations received by CSW, which also varied across the regions but less than was seen among MSM: 45 to 147 vaccinations per 100,000 women. Most first vaccinations of MSM were given at the public health services or STI clinics (ranging from 67.3 to 89.0% across the regions). More variation between the regions was seen in the location of first vaccination for CSW; in this group the percentage vaccinations given at outreach locations varied between 30.0 and 77.0% across the regions.

**Table 3:** Number and location of first vaccinations of the selective HBV vaccination programme for behavioural risk-groups by region, The Netherlands, 2009–2013.

	Brabant/Zeeland	Limburg	Noord	Noord-Holland	Oost	Utrecht	Zuid-Holland Noord	Zuid-Holland Zuid
	n = 3,065	n = 1,899	n = 1,756	n = 8,453	n = 3,899	n = 1,201	n = 2,194	n = 3,030
	N(%)	N(%)	N(%)	N(%)	N(%)	N(%)	N(%)	N(%)
No of 1st vaccinations								
MSM	2,426 (79.2)	1,222 (64.3)	954 (54.3)	6,449 (76.3)	3,033 (77.8)	831 (69.2)	1,431 (65.2)	2,446 (80.7)
CSW	639 (20.8)	677 (35.7)	802 (45.7)	2,004 (23.7)	866 (22.2)	370 (30.8)	763 (34.8)	584 (19.3)
No of 1st MSM vaccinations per 100,000 men*								
	171.6	219.6	111.8	424.6	194.9	138.3	281.7	198.6
No of 1st CSW vaccinations per 100,000 women*								
	45.0	119.6	93.0	128.4	54.7	58.9	147.0	46.1
Location of 1st vaccination MSM								
PHS/STI	1,886 (77.7)	1,088 (89.0)	729 (76.3)	4,935 (76.5)	2,508 (82.7)	708 (85.2)	963 (67.3)	1,772 (72.4)
Outreach	513 (21.1)	133 (10.9)	225 (23.6)	1,109 (17.2)	364 (12.0)	109 (13.1)	468 (32.7)	485 (19.8)
Other	27 (1.1)	1 (0.1)	1 (0.1)	405 (6.3)	160 (5.3)	14 (1.7)	0 (0.0)	189 (7.7)
Location of 1st vaccination CSW								
PHS/STI	379 (59.3)	466 (68.8)	289 (36.0)	272 (13.6)	360 (41.6)	82 (22.2)	217 (28.4)	286 (49.0)
Outreach	210 (32.9)	203 (30.0)	492 (61.3)	1,281 (63.9)	486 (56.1)	285 (77.0)	546 (71.6)	285 (48.8)
Other	50 (7.8)	8 (1.2)	21 (2.6)	451 (22.5)	20 (2.3)	3 (0.8)	0 (0.0)	13 (2.2)

MSM = men who have sex with men; CSW = commercial sex workers; PHS = public health centre; STI = sexually transmitted infections clinic

*Number of men or women is based on the average population 2009–2013 in that specific region

## Discussion & Conclusion

We used epidemiological data from notified acute hepatitis B cases between 2009 and 2013 together with molecular data and data on preventive activities, to study the regional differences in incidence, transmission and preventive activities in the Netherlands. We found that in the rural border areas the incidence of acute HBV infection was higher, most patients got infected through male homosexual contact or were men with an unknown transmission mode, and that patients were genetically more clustered, suggesting more ongoing transmission in these areas. In the more inland and more densely populated areas of the Netherlands, heterosexual transmission was the most common transmission mode and HBV sequences of patients were genetically less clustered, suggesting more single introductions in these areas. In addition, two statistically significant spatio-temporal clusters were identified (one in the southwest and one in the northeast of the Netherlands), but only the patients in the north-eastern spatio-temporal cluster statistically differed from the other patients in the country with regard to age, sex, ethnicity and genetic clustering. Finally, considering preventive activities, we found that the lowest rate of number of vaccinations among MSM per 100,000 men was seen in the northern region, which also showed the highest incidence among men.

The higher incidences in the rural border areas, specifically in the northeast, suggest that transmission of acute hepatitis B is not yet controlled in these areas. We found that it is mainly a problem among MSM: more ongoing transmission and relatively low vaccination rates. There might be several reasons for the higher incidences and lower vaccination rates among MSM in these areas. First, it could be possible that it is harder for the public health services to reach MSM in these areas for vaccination, since it could be the case that in these areas more men did not disclose their homosexuality. We observed that in the more rural areas of the Netherlands the percentage of men with an ‘unknown’ transmission route is relatively higher compared to the urban areas. This could be an indication of lower disclosure rates in these areas. If men do not openly come out for their homosexual contacts, they might also not identify themselves as being gay. This could lead to a low perceived risk of acquiring acute hepatitis B infection, which was shown to be the most important reason for opting out of the vaccination programme in the Netherlands [13,14]. Another explanation for the higher incidences among MSM in the rural areas is that MSM in the rural areas might have different patterns of risk behaviour than MSM in urban areas [15]. Finally, since it concerns rural areas, the distance to reach the public health services to get a vaccination can be larger than in the urban areas. This can therefore be another barrier for MSM to get vaccinated, and therefore explain the higher incidences in these areas.

The major strength of this study is the comprehensive approach of addressing this research question, by making use of both epidemiological and molecular sequence data. These different types of data are usually analysed separately, but we attempted to link and combine these data types and databases as much as possible, to get a complete overview of the hepatitis B situation in the Netherlands of the last five years.

There are several limitations of our study. First, although the notification of acute hepatitis B is obligatory in the Netherlands, it is likely that not all cases are included in this study since asymptomatic infections are not captured in the surveillance system. In addition, there is likely to be some underreporting, albeit less now that both laboratories and clinicians have to report cases. However, it can be expected that this missing of cases is randomly distributed throughout the country, which therefore will not influence our results. The second issue is that only around sixty per cent of all notified cases had a sample available for sequencing. We found that the percentage of samples available for sequencing was not distributed evenly across the regions, which could have been problematic if certain regions specifically selected certain cases to send in for sequencing. However, we could not detect this selection bias, and we can therefore assume that the sequenced samples reflect the patient populations in the regions. Finally, we calculated the rate of first vaccinations per 100,000 men or women (depending on the risk group) by region. This is a very crude rate since it assumes that the percentage of MSM or sex workers is distributed evenly throughout the regions, which is probably not true. Since it is not known how many MSM or CSW live in each region, our calculation provides currently the best possible estimate. In addition, MSM not always get their vaccination in the region they live, which could also influence this estimate.

Since 2011, hepatitis B vaccination in the Netherlands is part of the national vaccination programme for infants. It is expected that this will reduce the incidence of hepatitis B over what has been achieved by selective vaccination [6,16]. However, since most hepatitis B is transmitted sexually in the Netherlands, it will take several decades for this to be observed [6]. Therefore, it is of continued importance to put effort in further reducing transmission by improving or better targeting preventive activities. The results of this study made clear that the attention with regard to preventive activities should focus more on the MSM and not-yet-identified MSM in the rural areas. We recommend to further examine the reasons for the high incidences among MSM in these areas. Furthermore, it can be worthy to strengthen the outreach vaccination programme to overcome the distance barrier for vaccination. Finally, since the higher incidence regions are mostly located alongside the border with Germany, it might be useful to cooperate with our neighbouring country to further reduce the incidence in these areas.

To conclude, this study showed that regional differences in HBV epidemiology were present in the Netherlands. Rural border regions showed higher incidences and more ongoing transmission, mainly among MSM, than the more populated inland areas. Therefore, further preventive measures should focus more on these regions.
